# Clinical Reports

**Published:** 1877-02

**Authors:** 


### CLINICAL REPORTS.

### ATLANTA MEDICAL COLLEGE MEDICAL CLINIC.

### SERVICE OF J. G. WESTMORELAND, M.D.

ATLANTA, January 5, 1877.

GENTLEMEN : The history just given by this colored man is that he is 33 years old, and that eight months ago, after having contracted "a bad cold," cough ensued, and has continued to the present time. As you have observed, he complains of pain in left side of the chest, and says he lies more comfortably on the right side. From the patient's statements we iufer that the cough is more frequent and expectoration more copious during the night. The expectorated matter he now exhibits, consists mainly, I think, of tenaceous mucus. The character differs from this, probably, when at night a larger quantity is discharged.

His appearance, as you perceive, indicates emaciation and general debility. His pulse numbers about 115, and his breathing seems hurried. These symptoms are, however, doubtless increased by the effort of walking up stairs.

Percussion gives nearly normal sound on the left side of the chest, and decided dullness on the right, toward the upper portion, as you doubtless perceive, at the distance you are from the patient. Auscultation of the respiration gives pretty fair, though not perfect vesicular murmur in the left lung, while in the upper portion of the right it is barely perceptible. One not at all experienced in auscultating the respiration might conclude that breathing is perfect in the right lung; for the sound is equally loud and distinct as on the left side, but of different quality. Bronchial respiration is very clear on the right side, and by experience alone can it be distinguished from vesicular murmur.

In auscultation of the voice I find material difference in the lungs. While, by placing the ear on the left side, and the patient induced to talk, no very unusual sound is heard, the right side under the same circumstances gives a very peculiar penetrating sound. This is called bronchophony.

Now, gentlemen we have the history and present symptoms of the case before us. How are these to be reconciled with each other, and what pathological condition is to be determined by them? You remember the man complains of pain in the *left* side of the chest, and that he cannot lie comfortably on the left side. It would be very difficult to convince him that his left lung is sound, and perhaps some of you are at a loss how to reconcile these symptoms with the physical signs we have discovered by auscultation and percussion. In this case, then, you have proof of the very great importance of physical means of diagnosis.

In the first place I desire to call your attention to the fact that disease of the lung substance, unconnected with other structures, does not produce pain. This man feels pain or an uncomfortable sensation in the sound side, as I have frequently observed in others. This I attribute to the over-exertion of that lung, being taxed with a large proportion of the breathing, since the right fails to perform its part. Not being able to lie on the left side greatly strengthens the opinion entertained by the patient, that his disease is located in this part, but when viewed in the light of science an exactly opposite conclusion will be arrived at. The diseased lung, whether engorged with blood as in pneumonia, or made solid by the deposit of tuberculous matter, is much heavier than in a normal condition. Now, when the patient lies on the sound side, gravitation of the solid heavy lung, tends to press upon and obstruct the breathing of the already over taxed organ. When the pleura is principally involved in disease, the reverse of this will be found. In that case the diseased part must be uppermost in order to comfort. As great pain is the result of the slightest touch or movement when the pleura is inflamed, gravitation of the lung, whether heavier than usual or not, toward the diseased membrane, gives pain. So you will remember that in pluerisy the patient lies better on the sound, and in pneumonia on the diseased side.

The symptoms of unnaturally distinct bronchial respiration and bronchophony, in asucultation of the respiration and voice in the right side of this patient, depend upon the more ready and perfect conduction of sound through a solid than spongy medium. The voice through a healthy lung is not heard so distinctly by the ear applied to the chest as that receiving the sound through the air, but when the lung is made solid by deposit or engorgement, the transmission is so perfect that high-toned sounds of the voice are painfully penetrating to the applied ear. So, also, the sound of air passing along the bronchial tubes posterior to the solid lung are heard much more distinctly through it than when the healthy spongy structure intervenes.

Other facts must be considered in order to arrive at a proper conclusion in this case. You remember that this man contracted a cold eight months ago, which was accompanied and followed by a cough. I inquired whether he was in bed sick at that time, with the view of deciding first of all whether the disease took its origin in pneumonia. Finding that such condition was not probable, I relied upon the fact that tubercular deposit is more likely to occur in the upper portion or apex of the lung, to settle me in the opinion that the *right* lung is solid, as you discover in its upper portion, from the deposit of tuberculous matter, and that it probably existed before the attack of *bad cold.* If the present condition had resulted from pneumonia or bronchitis, the base of the lung would be, at least, equally implicated. I would be pleased to find convincing evidences that it is chronic pneumonia resulting from an acute attack. In that case we would be saved the difficult task of preventing the continued deposit of tubercles in the lung. Since, however, we have no such evidences, and since we are expected to do more than we are sometimes able to accomplish, notwithstanding the unfavorable prognosis we are compelled to make, I shall apply myself to the task as best I can.

The objects to be accomplished, or the indications to be filled are :

1st, to allay the irritation in the lung, and perhaps the bronchia upon which the cough depends, and insure rest and sleep at night; 2d, to give an increased amount of vitality or nervous power; 3d, to give increase of heat and nutrient material; and 4th, to arrest any chronic inflammation or ulceration that may exist in the lungs or broncia.

For the first, opium will answer well, not only to give relief from the disturbing irritation, but to afford that general nervous vigor so important in such condition. I therefore prescribe one-fourth grain morphine morning and night for a few days. In addition to this and for the accomplishment of these first objects, I direct the use of two fluid-ounces of good rye whisky three times a day. Croton oil rubbed on the right side of the chest in front will afford suituable counter irritation. This should be applied two or three times a day until a full crop of pustules is formed, and repeated as they disappear. The most nutritious and oily food is especially enjoined in order to the production of heat and the highest degree of nutrition. These are necessary to invigorate to a degree above the point at which, in strumous subjects, the production of tuberculous matter occurs. Our next prescription will be intended to affect locally the diseased surface of the bronchia and lungs, being carried to affected parts by inspiration. Iodine in the form of fumes taken by inhalation finds its way to all parts of the respiratory apparatus where air goes, and has local alterative or cathartic action not inferior to nitrate of silver and other articles of this class. A small wide mouthed vial makes a complete inhaler for this purpose. When heated, a scale of iodine dropped into it will instantly assume the form of fumes, and may be inhaled without difficulty. This should be taken once a day, for some two or three weeks, and then repeated at intervals of two or three days. In order to prevent excessive irritation and increase of the annoying cough from the inhalation, the iodine should be inhaled abont an hour after an opiate has been taken.

By this means iodine in any desired degree of concentration may be carried to any part of the respiratory organs receiving air, and although somewhat unpleasant sensation exists for a few moments, no fears need be entertained of injurious results, howevcr concentrated the fumes.

Nitrate of silver, carbolic acid and other remedies of the kind would doubtless answer the purpose of local alterative action very well, but they are not subject to forms of preparation in which they can be introduced to the minute bronchia and lungs. Powdered substances are likely to adhere when coming in contact with moist surfaces, and would not pass readily into the bronchia. Vapor carries such articles only as are volatile, and the inhalation of watery vapor from solutions of various non-volatile substances amounts to very little, so far as any local impression is concerned.

